# High and low inferior mesenteric artery ligation in laparoscopic low anterior rectal resections: A retrospective study

**DOI:** 10.3389/fsurg.2022.1027034

**Published:** 2023-01-13

**Authors:** Jun Yu, Yi Chen, Tong Li, Bo Sheng, Zhuo Zhen, Chang Liu, Jianbo Zhang, Qian Yan, Peng Zhu

**Affiliations:** ^1^ Department of Gastrointestinal Surgery, The Second Affiliated Hospital of Chongqing Medical University, Chongqing, China; ^2^ Health Management Center, The Second Affiliated Hospital of Chongqing Medical University, Chongqing, China

**Keywords:** rectal cancer, high ligation, low ligation, inferior mesenteric artery (IMA), anastomotic leakage (AL)

## Abstract

**Backgroud:**

The high or low inferior mesenteric artery (IMA) ligation in rectal cancer remains a great debate. This study retrospectively discussed the outcomes of the perioperative period, defecation and urinary function and long-term prognosis in rectal cancer patients with high or low IMA ligation.

**Methods:**

This study enrolled 220 consecutive rectal cancer cases, including 134 with high IMA ligation and 86 with low ligation. A comparison between the two groups was made for anastomotic leakage, low anterior resection syndrome (LARS), international prostate symptom score (IPSS), 5-year disease-free survival (DFS) and 5-year overall survival (OS).

**Results:**

Low-ligation group had a longer operative time, and larger intraoperative blood loss. No significant difference was noted in anastomotic leakage incidence. In multivariable analysis, the male gender and tumor located at the lower rectum were identified as risk factors for anastomotic leakage. No significant differences were observed between groups in their LARS and IPSS questionnaire responses. The high-ligation vs. the low-ligation 5-year OS and DFS were 78.3% vs. 82.4% and 72.4% vs. 76.6%, respectively, which were not statistically different.

**Conclusion:**

The ligation level of the IMA had no significant effect on the anastomotic leakage incidence, defecation, urinary function, and long-term prognosis.

## Introduction

Currently, the incidence and mortality attributed to colorectal cancer both rank third, with patients becoming younger in average age (1). Surgical treatment is the mainstay for rectal cancer, amongst which low anterior resections are most valued. In domestic practice and overseas, there remains debate about the position of the inferior mesenteric artery (IMA) ligation during surgery (2–5). High ligation refers to a ligation located 1 to 2 cm away from the abdominal aorta origin, without the left colic artery preservation. In contrast, low ligation refers to a ligation at the left colic artery region, with dissection of the lymph nodes at IMA root and preservation of the left colic artery (6–9). Some scholars supported the use of high ligation mainly for two reasons: on the one hand, high ligation allows a sufficient length of the proximal free colon, which ensures a situation free of tension for colonic anastomosis; on the other hand, high ligation raises the lymph node yield, and improves the precision of disease staging, in the way of allowing lymph nodes dissection at IMA root to the maximum (10–13). However, according to some scholars, high ligation is at the cost of abandoning proximal colonic blood perfusion and declining anastomotic blood supply, which might increase the incidence of anastomotic leakage and lead to colon necrosis due to ischemia in severe cases (14–17). Researchers have not yet discovered if high ligation contributes to a higher lymph node yield in rectal resections, while it shows no superiority to low ligation regarding long-term oncologic prognosis (8, 18, 19).

In addition, high ligation with complete lymph node IMA root dissection may damage the inferior epigastric plexus, which governs defecation, urination, and sexual function. The results are conflicting on the effect of IMA ligation level on defecation and urinary function (20, 21).

This study retrospectively discussed the outcomes of the perioperative period, defecation, urinary function, and long-term prognosis in rectal cancer patients who had a laparoscopic lower rectal anterior resection with high or low IMA ligation.

## Methods

### Patients

This retrospective study involved 220 participants treated with radical laparoscopic for rectal cancer in the Second Affiliated Hospital of Chongqing Medical University between January 2014 and May 2016. Inclusion criteria were (1) Distance between tumor edge and the anal verge ≤15 cm; (2) Preoperative colonoscopy confirmed rectal tumor, and the pathological tissue biopsy revealed adenocarcinoma; (3) Preoperative chest and abdomen computer tomography (CT) and pelvic MRI confirmed locally progressive tumor; (4) Intraoperative sigmoid-rectal end-to-end anastomosis with a double stapling apparatus. Exclusion criteria were (1) Stage IV carcinoma with distant or peritoneal metastases before or during operation; (2) Emergency patients complicated with bleeding, perforation, and intestinal obstruction; (3) Patients with Hartmann's or abdominoperineal resections; (4) Multiple colorectal cancers. The patient whose preoperative MRI suggested T3–4 or N+ was treated with concurrent radiotherapy and chemotherapy. The long course of preoperative radiotherapy consisted of 50.4 Gray in 25 to 28 fractions, five times per week, over five weeks. Oral 5-fluorouracil (Capecitabin, Xeloda®, 825 mg/m^2^/day, twice a day, five times a week, for five weeks) was administered in conjunction with a long course of radiotherapy. After concurrent chemoradiotherapy, surgery was performed six weeks later. The hospital ethics committee approved this research.

### Surgical procedure

All surgeries were performed by five professors, each with 20 years of experience in gastrointestinal surgery, who decided on the ligation level of IMA according to the intraoperative situation and personal opinions. IMA was ligated 1 to 2 cm distant from the aorta origin, with clearance of lymph nodes at the root in the high-ligation cases. IMA was ligated in the lower part of the left colic artery following lymph node dissection at the root in the low-ligation cases. Following the sigmoid-rectal end-to-end anastomosis completion, one drainage tube was placed around the pelvic anastomosis and passed out through the abdominal wall. The surgeon decided the level of IMA ligation, whether to perform a preventive ileostomy, and whether to place an anal canal.

### Postoperative management

Training for bladder function was arranged by clamping the urinary catheter on day 2 after the operation, followed by removing the urinary catheter. The anal canal was removed on day 5. The abdominal drainage tube was removed on day 7 in case of the absence of anastomotic leakage. Ileostomy closure was arranged 1–3 months after the operation for patients with a preventive ileostomy.

### Postoperative complications and pathology

The postoperative complications were categorized as per the Clavien-Dindo method. Mild and serious complications were determined if the Clavien-Dindo classification was ≤II or ≥III, respectively (22). Anastomotic leak was defined as fecal flow through the abdominal drainage tube or signs of peritonitis, and the presence of an anastomotic leak was confirmed by abdominal CT. The anastomotic leak was graded following the International Study Group of Rectal Cancer grading: grade A, no special treatment required; grade B, active treatment required without reoperation; grade C, operative treatment (23). Postoperative tumor TNM pathological staging followed the American Joint Committee on Cancer (AJCC) 8th edition. An involved circumferential resection margin (CRM) was defined as ≤1 mm between the margin of deepest tumor infiltration and the surgical resection margin.

### Postoperative adjuvant chemotherapy

Stage I patients were followed up regularly, stage II patients received adjuvant oral 5-FU-based chemotherapy (capecitabine), and stage III patients received Xelox (capecitabine plus oxaliplatin).

### Functional evaluation

Low anterior resection syndrone (LARS) score was utilized to assess bowel function. LARS scoring questionnaire, consisting of five questions about liquid stool and flatus incontinence, stools clustering, bowel frequency, and fecal urgency, was scored from 0 to 42. The patients were classified as no LARS, minor LARS, and major LARS when the score was 0–20, 21–29, and 30–42 points, respectively (24). The international prostate symptom score (IPSS) for urinary function consisted of seven items: urgency, frequency, nocturia, weak stream, intermittency, incomplete emptying, and straining (25). IPSS was classified as mild, moderate, or severe when the score was 0–7, 8–19, or 20–35, respectively. Prior to and at 6 and 12 months after surgery, the patients were given a questionnaire. At 6 and 12 months after ileostomy closure, patients with ileostomies completed questionnaires to assess bowel function.

### Follow up

After surgery, all patients were followed up every six months for the first three years and then annually for three to five years. Follow-up visits, conducted in the clinic and by telephone, included a physical examination, carcinoembryonic antigen measurement, CT of the chest and abdomen, colonoscopy, and completion of a questionnaire. If patients were found to suffer recurrent metastasis for the follow-up period, the location and point in time of recurrent metastasis were recorded. Patients with recurrent metastasis were reexamined every three months, assessing serum carcinoembryonic antigen and CT of the chest and abdomen.

### Statistical analysis

The study statistical analyses were done using macOS IBM SPSS Statistics 26.0. Comparing categorical variables and continuous data among different groups was done *via* Chi-square or Fisher's exact tests. Relying on the distribution, the continuous data were evaluated with an independent *t*-test or Mann–Whitney *U* test. Univariate and multivariate logistic regression assessed the risk factors for anastomotic leakage. *p* < 0.100 variables were included in the multivariable analysis. The 5-year OS and DFS were analyzed by Kaplan-Meier curves, and, to verify the groups' significant differences, a log-rank test was done. *p* < 0.05 was regarded as statistically significant.

## Results

### Patient characteristics

Table 1 shows all patients' baseline and clinical characteristics. No statistically significant differences were found between patients who were treated with high ligation and those who underwent low ligation for gender, age, ASA stage, BMI, tumor location, neoadjuvant chemoradiotherapy, history of abdominal surgery, diabetes, coronary heart disease, and hypertension between the two groups (*p* > 0.05).

**Table 1:** Patients’ baseline and clinical characteristics.

	High ligation (*n* = 134)	Low ligation (*n* = 86)	*P* value
Age (years)	63.6 ± 6.9	65.1 ± 6.8	0.110
Gender			0.442
Male	71 (53.0%)	41 (47.7%)	
Female	63 (47.0%)	45 (52.3%)	
BMI (kg/m2)	24.7 ± 2.5	25.0 ± 2.1	0.242
ASA			0.648
I	47 (35.1%)	25 (29.1%)	
II	69 (51.5%)	48 (55.8%)	
III	18 (13.4%)	13 (15.1%)	
Tumor location			0.302
Upper rectum	91 (67.9%)	64 (74.4%)	
Lower rectum	43 (32.1%)	22 (25.6)	
Neoadjuvant therapy	27 (18.8%)	18 (20.9%)	0.687
History of abdominal surgery	39 (29.1%)	17 (19.8%)	0.121
Diabetes	22 (16.4%)	13 (15.1%)	0.797
Coronary heart disease	27 (20.1%)	18 (20.9%)	0.889
Hypertension	31 (23.1%)	26 (30.2%)	0.241

### Surgical data and postoperative complications outcomes

Table 2 shows the surgical outcomes and complications. The high ligation group had a shorter operation time than the low ligation group (184.6 ± 14.4 min vs. 190.7 ± 16.4 min, *p* = 0.004). Intraoperative blood loss in the high ligation group was significantly higher than in the low ligation group (91.2 ± 21.8 ml vs. 84.3 ± 24.5 ml, *p* = 0.037). No statistical differences were shown in conversion to open surgery, splenic flexure mobilization, preventive ileostomy, indwelling anal canal, time to first flatus, and hospital stay (*p* > 0.05). The incidence of postoperative complications in the high and low ligation groups was 25.4% and 20.9%, respectively, and no significant difference was observed (*p *= 0.449). The anastomotic leakage in the high and low ligation groups was 10.4% (14 patients) and 8.1% (7 patients), respectively, with no significant difference (*p *= 0.570). Reoperation occurred in the high and low ligation groups at 3.7% and 3.5%, respectively (*p *= 0.534). The 30-day after surgery mortality in the high and low ligation groups were two and one case, respectively, which was not significantly different.

**Table 2:** Surgical data and postoperative complications.

	High ligation (*n* = 134)	Low ligation (*n* = 86)	*P* value
Conversion to open surgery	5 (3.7%)	2 (2.3%)	0.708*
Operative time (min)	184.6 ± 14.4	190.7 ± 16.4	0.004
Intraoperative blood loss (ml)	84.3 ± 24.5	91.2 ± 21.8	0.037
Splenic flexure mobilization	20 (14.9%)	18 (20.9%)	0.250
Preventive ileostomy	17 (12.7%)	13 (15.1%)	0.608
Indwelling anal canal	94 (70.1%)	61 (70.9%)	0.695
Time to first flatus (day)	3.6 ± 1.0	3.6 ± 1.1	0.539
Hospital stays (day)	10.2 ± 2.6	10.0 ± 3.0	0.720
Postoperation complications	34 (25.4%)	18 (20.9%)	0.449
Dindo-Clavien classification			0.734
Mild	23 (17.2%)	13 (15.1%)	
Severe	11 (8.2%)	5 (5.8%)	
Incision infection	5 (3.7%)	4 (4.7%)	0.739*
Intestinal obstruction	3 (2.2%)	1 (1.2%)	1*
Diarrhea	2 (1.5%)	1 (1.2%)	1*
Urinary retention	5 (3.7%)	2 (2.3%)	0.708*
Pneumonia	4 (3.0%)	2 (2.3%)	1*
Anastomotic bleeding	1 (0.7%)	1 (1.2%)	1*
Anastomotic leakage	14 (10.4%)	7 (8.1%)	0.570
Leakage grade			0.704*
A	2 (1.5%)	2 (2.3%)	
B	5 (3.7%)	2 (2.3%)	
C	7 (5.2%)	3 (3.5%)	
Reoperation	8 (3.7%)	3 (3.5%)	0.534*
Overall 30-day mortality	2 (1.5%)	1 (1.2%)	1*

* Refers to Fisher's exact test.

### Anastomotic leakage risk factors

The male gender, neoadjuvant therapy, and the lower rectum tumors were considerably related to anastomotic leakage incidence, as revealed by univariable analysis. The male gender and the lower rectum tumors were considered anastomotic leakage risk factors, as the multivariable analysis revealed (Table 3).

**Table 3:** Risk factors for anastomotic leakage.

	Anastomotic leakage		Univariable analysis		Multivariable analysis	
	Yes (*n* = 21)	No (*n* = 199)	*P*	OR (95%CI)	*P*	OR (95%CI)
**Gender**						
Male	17	96	0.011	4.333 (1.399–13.418)	0.035	3.451 (1.091–10.919)
Female	4	104		1 (reference)		1 (reference)
**Age**						
>65	11	84	0.492	1.381 (0.550–3.467)	–	–
<65	10	116		1 (reference)	–	–
**Neoadjuvant therapy**						
Yes	8	38	0.038	2.296 (0.858–6.143)	0.129	1.651 (0.599–4.545)
No	13	162		1 (reference)		1 (reference)
**Tumor location**						
Lower rectum	12	54	0.012	3.305 (1.298–8.414)	0.047	2.628 (1.011–6.828)
Upper rectum	9	146		1 (reference)		1 (reference)
**Diverting ileostomy**						
Yes	4	27	0.852	0.884 (0.310–4.119)	–	–
No	17	173		1 (reference)	–	–

### Pathological outcomes

Table 4 lists the pathological results summary. The tumor size, proximal margin, and distal margin were measured without differences between the two groups (*p *> 0.05). The number of lymph nodes harvested in the high and low ligation groups was 16.3 ± 2.8 and 15.5 ± 2.4, respectively (*p *= 0.053). No significant difference was observed between the two groups in the number of positive lymph nodes (*p *= 0.493). No statistical differences were identified between the two groups in CRM, neural invasion, vascular invasion, degree of differentiation, pN stage, pT stage, and pTNM stage (*p* > 0.05; Table 4).

**Table 4:** Pathological data.

	High ligation (*n* = 134)	Low ligation (*n* = 86)	*P* value
Tumor size (cm)	3.8 ± 1.4	3.6 ± 1.5	0.170
Proximal margin (cm)	9.5 ± 2.2	8.9 ± 2.5	0.064
Distal margin (cm)	2.2 ± 1.1	1.9 ± 1.1	0.141
CRM			0.985
Negative	123 (91.8%)	79 (91.9%)	
Positive	11 (8.2%)	7 (8.1%)	
Neural invasion	21 (15.7%)	10 (11.6%)	0.400
Vasculature invasion	18 (13.4%)	11 (12.8%)	0.891
Degree of differentiation			0.534
High	95 (70.9%)	55 (64.0%)	
Medium	24 (17.9%)	18 (20.9%)	
Low	15 (11.2%)	13 (15.1%)	
pT stage			
T1	8 (6.0%)	6 (7.0%)	0.612
T2	19 (14.2%)	13 (15.1%)	
T3	58 (43.3%)	43 (50.0%)	
T4	49 (36.6%)	24 (27.9%)	
pN stage			0.749
N0	96 (71.6%)	65 (75.6%)	
N1	29 (21.6%)	15 (17.4%)	
N2	9 (6.7%)	6 (7.0%)	
pTNM			0.803
I	27 (20.1%)	19 (22.1%)	
II	69 (51.5%)	46 (53.5%)	
III	38 (28.4%)	21 (24.4%)	
Total number of lymph nodes harvested	16.3 ± 2.8	15.5 ± 2.4	0.053
Positive number of lymph nodes harvested	0.8 ± 2.0	1.0 ± 2.2	0.493

### Functional outcomes of LARS and IPSS questionnaires

The functional outcomes of LARS and IPSS questionnaires are shown in Table 5. No significant differences were observed before surgery, or 6 and 12 months following surgery, in both LARS and IPSS questionnaire responses between groups.

**Table 5:** Function outcomes of LARS and IPSS.

	High ligation	Low ligation	*P* value
Preoperational LARS grade	134	86	0.275
No	72 (53.7%)	49 (64.5%)	
Minor	45 (33.6%)	18 (23.7%)	
Major	17 (12.7%)	9 (11.8%)	
6-month LARS grade	115	72	0.689
No	23 (20.0%)	12 (16.7%)	
Minor	55 (47.8%)	39 (54.2%)	
Major	37 (32.2%)	21 (29.2%)	
12-month LARS grade	93	64	0.706
No	32 (34.4%)	18 (28.1%)	
Minor	46 (49.5%)	35 (54.7%)	
Major	15 (16.1%)	11 (17.2%)	
Preoperational IPSS grade	134	86	0.435
Mild	51 (38.1%)	40 (46.5%)	
Moderate	46 (34.3%)	24 (27.9%)	
Sever	37 (27.6%)	22 (25.6%)	
6-month IPSS grade	115	72	0.699
Mild	27 (23.5%)	19 (23.2%)	
Moderate	47 (40.9%)	38 (46.3%)	
Sever	41 (35.7%)	25 (30.5%)	
12-month IPSS grade	93	64	0.171
Mild	31 (33.3%)	17 (26.6%)	
Moderate	34 (36.6%)	33 (51.6%)	
Sever	28 (30.1%)	14 (21.9%)	

### Long-time oncologic prognosis

**Figure 1:**
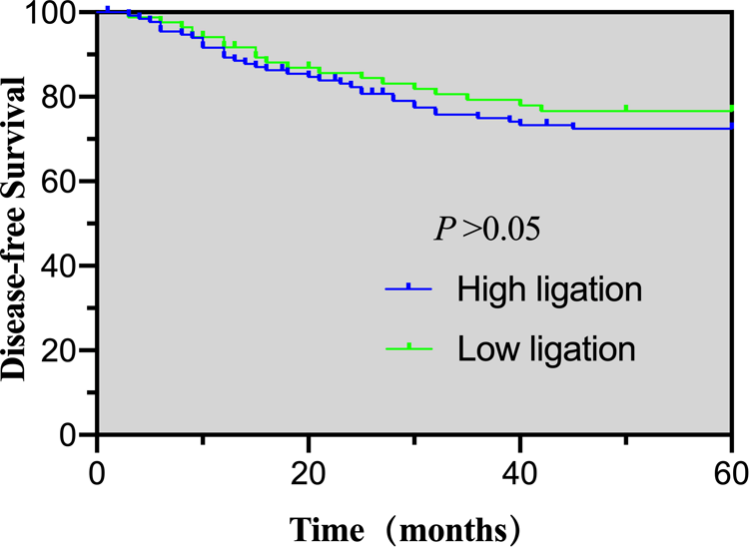
5-year disease-free survival of the high- and low-ligation groups.

**Figure 2:**
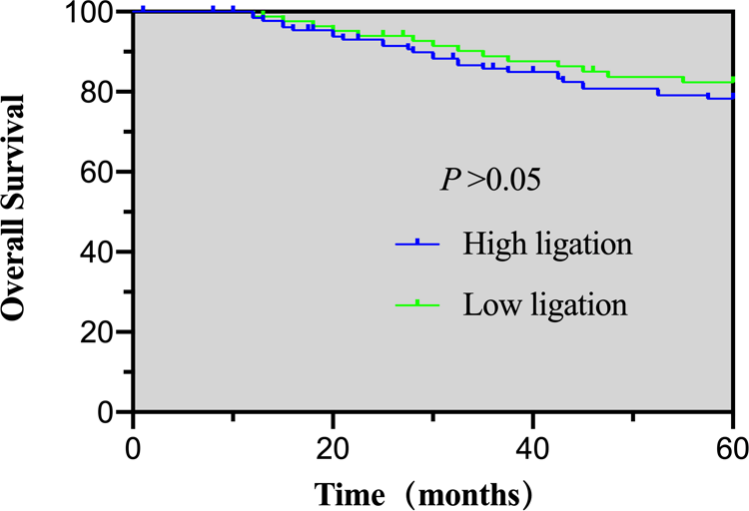
5-year overall survival of the high- and low-ligation groups.

The follow-up rate at five-years was 90.5%, with 12 patients in the high ligation group and nine patients in the low ligation lost to follow-up. The occurrence of recurrent metastases in the high and low ligation groups was 26.5% vs. 22.4%, respectively, which was not statistically different (Table 6). The 5-year OS and DFS for the high and low ligation groups were 78.3% vs. 82.4% (*p* = 0.463), and 72.4% vs. 76.6% (*p* = 0.485), respectively, showing no statistical differences (Figures 1 and 2).

**Table 6:** Recurrent metastasis and long-time outcome.

	High ligation (*n* = 132)	Low ligation (*n* = 85)	*P* value
Recurrent metastasis	35 (26.5%)	19 (22.4%)	0.489
Liver metastasis	15 (11.4%)	8 (9.4%)	0.648
Pulmonary metastasis	10 (7.6%)	3 (3.5%)	0.220
Liver and pulmonary metastasis	8 (6.1%)	7 (8.2%)	0.538
Local recurrence	2 (1.5%)	1 (1.2%)	1*
5-year overall survival	78.3%	82.4%	0.463
5-year disease-free survival	72.4%	76.6%	0.485

* Refers to Fisher's exact test.

## Discussion

This study discloses that low ligation increases operation time and intraoperative blood loss compared to high ligation. Furthermore, no significant difference was observed between the high and low ligation groups in anastomotic leakage. The male gender and lower rectum tumors are anastomotic leakage risk factors. No significant differences were observed between the groups in oncologic outcomes, such as 5-year OS and 5-year DFS, as well as functional outcomes, such as bowel and urine functions.

The study revealed that the low ligation group had a longer operative time (184.56 ± 14.4 vs. 190.7 ± 16.4, *p* = 0.004) and more intraoperative blood loss (84.3 ± 24.5 vs. 91.2 ± 21.8, *p* = 0.037). Given that low-ligation works by lymph node clearance at IMA root to expose the left colic artery, on the premise of IMA safety, it is harder to run and requires more experienced surgeons. A recent study found that whether the left colic artery is preserved or not was independent of the operative time and intra-operative blood loss (5). Similarly, some meta-analyses showed that the left colic artery preservation would not increase the operative time and intra-operative blood loss (26, 27). However, Park et al. (8) reported that the low-ligation strategy contributed to a shorter operative time but was not superior in decreasing intraoperative blood loss. The discrepancy might be associated with the operative experience of the surgeons.

Anastomotic leakage is a high-risk complication during low anterior rectal resections, leading to a longer hospital stay and higher medical costs, as well as increasing the ileostomy rate and mortality. In the current research, the anastomotic leakage incidence in the high and low ligation groups were 10.4% and 8.1%, respectively, which was not statistically significant, and the male gender and the lower rectum tumor were considered risk factors. Several studies showed that the anastomotic leakage incidence during low anterior rectal resections would not be increased when high-ligation was applied, and gender and the distance from the tumor to the anus were major factors causing anastomotic leakage after operation (28, 29). The level of IMA ligation does not correlate with anastomotic leakage and must be selected according to factors, including the presence or absence of tension anastomosis (30). Additionally, it has been suggested that anatomical variants of the left colic artery should be of concern, as insufficient vascularization of the proximal colonic conduit in the absence of the left colic artery is also an important factor in the occurrence of anastomotic leakage (31). However, anastomotic blood perfusion remains one of the important factors affecting anastomotic healing. Intraoperative colonic perfusion was measured using laser Doppler flowmetry and was found to be slightly decreased in the high ligation group and slightly increased in the low ligation group, independent of blood pressure (16). Seike et al. (15) reported that, after clamping the IMA, the anastomotic blood flow of the proximal colon was significantly reduced, which was more evident in elderly men, along with a higher risk of anastomotic leakage. Other studies also demonstrated that low ligation could decrease the anastomotic leakage risk (32–34). Therefore, larger samples are required to further explore the relationship between anastomotic leakage and IMA ligation level in the future.

Bowel and urinary function were poor after rectal cancer surgery. A Japanese randomized controlled trial reported no significant differences between patients with high and low ligation, assessed at three months and one year postoperatively, on defecatory function, fecal incontinence quality of life scale defaecation self-assessment, or continence score (9). Defecation function, related to levels of IMA ligation resulting in different blood supply to the anastomosis, is also related to other factors, such as the denervated neorectum motility, rectal compliance, anal sphincter, and anastomosis level. Although neither group returned to preoperative IPSS levels, there was an improvement in IPSS at nine months after low ligation compared to high ligation (20). Park (8) reported no difference in bowel and urinary function between the two groups before surgery, three months after surgery, and 12 months after surgery. Similarly, this research revealed no significant statistical difference in LARS and IPSS between high and low ligation in preoperative, six months postoperative, and 12 months postoperative. The difference may be related to autonomic nerve injury during IMA peripheral lymph node dissection in our surgery.

The number of lymph nodes dissected during operation is vital for operation assessment, guiding postoperative adjuvant chemoradiotherapy and prognosis. Patients with lymph node metastasis are more likely to have tumor recurrence and experience a shorter survival time compared to those without metastasis (35). Research revealed no evidence showing the benefits of high-ligation in long-term prognosis, although it could get more lymph nodes dissected (36). Many current studies have suggested that there were no more lymph nodes dissected by high-ligation, and still, no superiority was demonstrated in long-term prognosis compared to low-ligation (37–41). There were no statistical differences between the total number of lymph nodes dissected and the number of positive ones. Moreover, the 5-year OS and 5-year DFS showed no evident differences between the two groups.

Several limitations remain in this study. First, this is a retrospective study involving a small sample size from a single institute, requiring larger-scale, multi-center, and randomized controlled trials for further validation. Second, selection bias might not be ignored. Finally, sexual functions, such as the international index of erectile function (IIEF-5), and the female sexual function index (FSFI), were not assessed in this study.

## Conclusions

The ligation level of IMA has no significant effect on the incidence of anastomotic leakage, defecation, urinary function, or long-term prognosis. However, larger randomized controlled trials are still required to further validate this result.

## Data Availability

The raw data supporting the conclusions of this article will be made available by the authors, without undue reservation.
